# The Influence of a Binder in a Composite Electrode: The Case Study of Vanadyl Phosphate in Aqueous Electrolyte

**DOI:** 10.3390/ma15249041

**Published:** 2022-12-17

**Authors:** Dragana Jugović, Miloš D. Milović, Tanja Barudžija, Maja Kuzmanović, Milica Vujković, Miodrag Mitrić

**Affiliations:** 1 Institute of Technical Sciences of SASA, 11000 Belgrade, Serbia; 2 “VINČA” Institute of Nuclear Sciences—National Institute of the Republic of Serbia, University of Belgrade, 11000 Belgrade, Serbia; 3 Faculty of Physical Chemistry, University of Belgrade, 11000 Belgrade, Serbia

**Keywords:** VOPO_4_·2H_2_O, aqueous batteries, binder, crystal structure refinement, electrochemical properties

## Abstract

Layered VOPO_4_·2H_2_O is synthesized by the sonochemical method. An X-ray powder diffraction is used to examine the crystal structure, while scanning electron microscopy is used to reveal the morphology of the powder. The crystal structure refinement is performed in the P4/*nmm*Z space group. The electrochemical intercalation of several cations (Na^+^, Mg^2+^, Ca^2+^, and Al^3+^) in saturated nitrate aqueous solutions is investigated. The most notable reversible activity is found for the cycling in aluminium nitrate aqueous solution in the voltage range from −0.1 to 0.8 V vs. SCE. During the preparation of the electrode, it is observed that the structure is prone to changes that have not been recorded in the literature so far. Namely, the use of conventional binder PVDF in NMP solution deteriorates the structure and lowers the powder’s crystallinity, while the use of Nafion solution causes the rearrangement of the atoms in a new crystal form that can be described in the monoclinic *P*2_1_/*c* space group. Consequently, these structural changes affect electrochemical performances. The observed differences in electrochemical performances are a result of structural rearrangements.

## 1. Introduction

The development of safe, durable, cheap, and environmentally friendly batteries is one of the most important challenges of modern electrochemistry. Hence, there is an interest in the research of aqueous multivalent ion batteries, such as calcium, magnesium, and aluminium aqueous batteries [1,2]. VOPO_4_·2H_2_O, with its layered structure, is a particularly interesting and promising material for cation intercalation [3,4,5,6,7,8,9,10,11,12,13]. Vanadium V^5+^ ion can be reduced to a +3-oxidation state, opening up the possibility of more than one electron reaction per transition metal. The layered structure of VOPO_4_·2H_2_O can intercalate various species (molecules or ions) that cause a change in the interlayer distance [14,15,16] or/and the exfoliation of the bulk structure into 2D sheets [3,14,17]. Various cations can intercalate within the VOPO_4_·2H_2_O structure by redox reactions in iodide solutions [18,19,20]. However, there are few reports on the electrochemical intercalation of different cations in aqueous electrolytes. Literature data mainly report the electrochemical intercalation of zinc [3,12,21,22,23,24] and aluminium ions [7,11] in aqueous electrolytes. The electrochemical measurements use a composite electrode comprising active mass particles, conductive additives, and a polymeric binder. Binders have an important effect on the performance of the cell by maintaining both the mechanical and electrical integrity of the electrode network [25]. However, the presence of a binder may influence the properties of the materials under examination to an unknown extent [26]. Poly(vinylidene fluoride) (PVDF) dissolved in N-methyl-2-pyrrolidone (NMP) is the most commonly used binder solution [27]. Due to both economic and healthcare issues connected to NMP, there is a need for less expensive and less hazardous aqueous-based solvents [28]. For that reason, another frequently used binder is Nafion (a sulfonated tetrafluoroethylene-based fluoropolymer-copolymer), which dissolves in aqueous-based solvents.

This work reports the electrochemical intercalation of several cations (Na^+^, Mg^2+^, Ca^2+^, and Al^3+^) in saturated nitrate aqueous solutions in sonochemically derived VOPO_4_·2H_2_O. The working electrodes were prepared with PVDF or Nafion as a binder. It is revealed that, regardless of the choice of the binder, aluminium exhibits the most notable electrochemical activity among other ions, holding promise for its potential application in aqueous batteries. An interesting observation was made, namely that the application of the binder influences the structure of the active material, something that has not been reported in the literature for vanadyl phosphate.

## 2. Materials and Methods

The sonochemical method was used for the synthesis of the VOPO_4_·2H_2_O powder. By following the experimental data given in ref. [10], the synthesis was performed within a short time. A high-intensity ultrasonic probe of 750 W (Vibracell sonicator VCX 750, Sonics & Materials Inc., Newtown, CT, USA) that operates at 20 kHz with a 13 mm Ti-alloy probe was used to sonicate the reaction mixture. The reaction mixture, consisting of 1 g of V_2_O_5_, 15.8 mL of 85% H_3_PO_4_, and 100 mL of water, was introduced to the ultrasound irradiation under ambient air for 20 min while working in a pulsed mode: 1 s with an ultrasound on followed by a rest for 1 s. The obtained yellow powder was washed with water and acetone and dried at 100 °C.

X-ray powder diffraction was performed on a Philips PW 1050 diffractometer (Amsterdam, The Netherlands) with Cu–Kα_1,2_ radiation (Ni filter) at room temperature. The diffraction data were collected in two manners: a scanning step width of 0.05° and 3 s time per step in a 2θ range of 10–70° and a scanning step width of 0.02° and 14 s per step in the 2θ range from 10–110° were applied for phase identification and for the crystal structure refinement, respectively.

SEM analysis was performed on a Mira XMU TESCAN electron microscope (Kohoutovice, Czech Republic).

The FTIR spectra of the samples were recorded at ambient conditions in the mid-IR region (400–4000 cm^−1^) with a Nicolet IS 50 FT-IR spectrometer (Waltham, MA, USA) operating in the ATR mode with a measuring resolution of 4 cm^−1^ and 32 scans.

The electrochemical measurements were conducted by using Vertex.One potentiostat/galvanostat (Ivium Technologies, Eindhoven, The Netherlands). The measurements were performed using four different aqueous electrolytes (saturated aqueous solutions of Al(NO_3_)_3_, Ca(NO_3_)_2_, NaNO_3_, and Mg(NO_3_)_2_). The working electrode was made from a slurry of active material, carbon black PBX51 (Cabot Corp., Boston, MA, USA), and a binder (PVDF, 2.4 wt% solution in N-methyl-2-pyrroliperformed or Nafion, 5 wt% solution in a mixture of lower aliphatic alcohols and water) mixed in a weight ratio of 70:20:10, respectively. The slurry was deposited on glassy carbon and then dried at 120 °C. Cyclic voltammetry (CV) measurements were performed in a three-electrode cell with platinum as a counter electrode and SCE (saturated calomel electrode; SI Analytics, Mainz, Germany) as a reference electrode. Galvanostatic measurement was performed in a two-electrode cell with glassy carbon as a counter electrode. Electrochemical impedance spectroscopy (EIS) measurements were performed within the frequency range of 10^5^ to 10^−2^ Hz with a perturbation amplitude of 5 mV. The impedance spectra were recorded at the potential of the cathodic peak at the CV curve.

## 3. Results and Discussion

### 3.1. The Structure and Morphology

X-ray powder diffraction data were used for both phase check and crystal structure refinement. The structure of the powder was refined in the tetragonal space group P4/*nmm*Z (No. 129) with the following crystallographic positions: vanadium ions occupy 2*c* [¼, ¼, z] octahedral crystallographic position; phosphorus ions are located at a tetrahedral position 2b [¾, ¼, ½]; there are four different crystallographic positions where oxygen ions are allocated: additional two 2c positions (denoted as O2 and O4), one 8i [¼, y, z] position (denoted as O1), and 2a [¾, ¼, 0] crystallographic position (denoted as O3). Crystal structure refinement was based on the Rietveld full-profile method [29] using the Koalariet computing program, based on the fundamental parameters convolution approach in diffraction line profile fitting [30].

**Figure 1:**
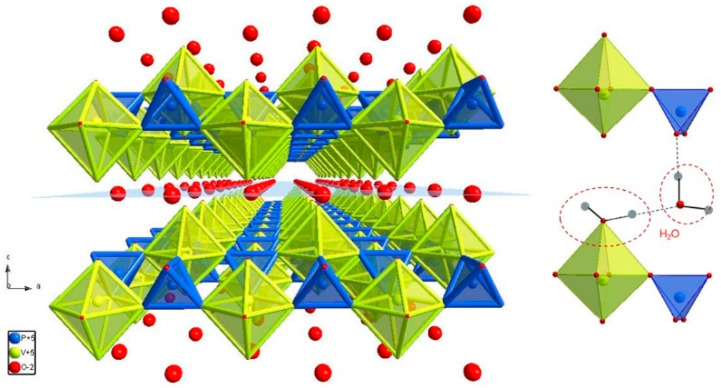
The polyhedral illustration of the structure of the VOPO_4_·2H_2_O created by the molecular and crystal structure visualization software Diamond 3.2 (**left**); the positions of water molecules (**right**).

**Figure 2:**
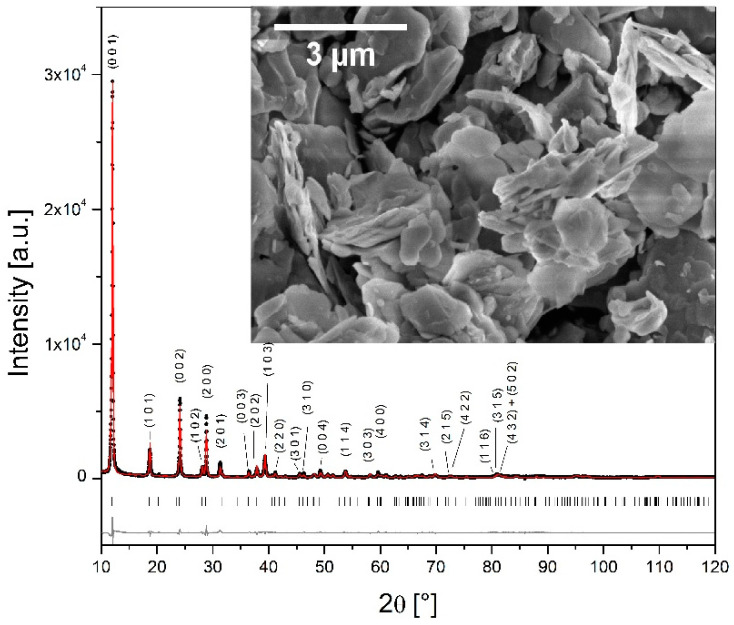
The observed (∙), calculated (-), and the difference between the observed and calculated (bottom) X-ray diffraction data taken at room temperature of VOPO_4_·2H_2_O powder. Vertical markers below the diffraction patterns indicate the positions of possible Bragg reflections. Inset: SEM micrograph of VOPO_4_·2H_2_O powder.

The structure (Figure 1) can be illustrated by infinite layers of VO_6_ octahedra connected to the PO_4_ tetrahedra. Within the layer, each VO_6_ octahedron is corner-connected to four PO_4_ tetrahedra via its equatorial oxygen ions (O1 site) in a zig-zag manner. Namely, the VO_6_ octahedron is distorted along the *c* axis and its apical oxygen ions (O2 and O4) alternate up and down relative to the layer. The water molecules are located between two layers. The interslab distance varies with the content of interlayered water. There are two kinds of water molecules (Figure 1): structural water, which is directly bonded to vanadium ion (at the O2 site), and crystal water, which is located between two VPO layers and contains oxygen O3 that belongs neither to VO_6_ octahedron nor PO_4_ tetrahedron [13,31]. The second water molecule forms hydrogen bonds with two PO_4_ groups of different layers [31]. The results of the refinement are given in Table 1 and Table 2, while its graphical representation is given in Figure 2. The lattice parameters (*a* = 6.214, *b* = 6.214, and *c* = 7.414 Å) are in good agreement with the literature data [10,13,28]. The value of the *c* parameter implies that the structure has two water molecules per formula unit [32]. Both refined and fixed fractional atomic coordinates were used to calculate all relevant bond distances given in Table 3. A significantly shorter V–O4 bond implies the presence of the [V=O]^3+^ cation [33].

SEM micrographs reveal platelet morphology (inset of Figure 2). Micron-sized particles show the lamellar assembly typical for layered structures.

**Figure 3:**
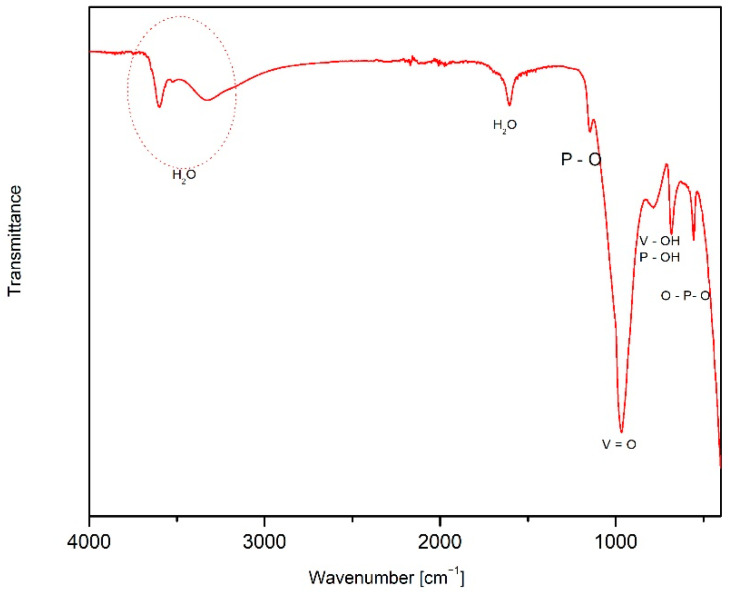
FTIR spectrum of the VOPO_4_·2H_2_O.

The FTIR spectrum of the as-synthesized powder (Figure 3) shows characteristic bands for VOPO_4_·2H_2_O [31]. Absorption bands that correspond to V-O and P-O stretching appear between 1200 and 600 cm^−1^, while O–V–O and O–P–O bending modes can be observed below 600 cm^−1^ [6,19]. The stretching O–H modes of water molecules can be seen from 3300 to 3600 cm^−1^, and bending bands are seen around 1600 cm^−1^. In the hydroxyl-stretching region, two observed bands can be assigned to two types of water molecules. The sharp one at 3600 cm^−1^ corresponds to the lattice water molecule, which is bound to the vanadium, while the hump at 3330 cm^−1^ is ascribed to the interlayer water molecule, which is not fully hydrogen-bonded and therefore less tightly bonded [31,33].

### 3.2. Behind the Scenes of Electrode Preparation

**Figure 4:**
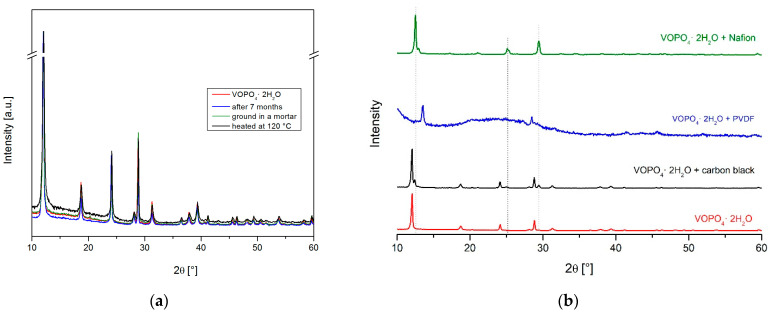
(**a**) XRD patterns of the VOPO_4_·2H_2_O powder after grinding in a mortar, drying at 120 °C, and ageing in the air for 7 months; (**b**) Comparison of the XRD patterns of the VOPO_4_·2H_2_O powder, its mixtures with carbon black (PBX51), and carbon black and a binder (PVDF or Nafion).

The electrochemical measurements are performed with the composite electrode comprising active mass particles, conductive additives, and a polymeric binder. Since there were indices that the active material, VOPO_4_·2H_2_O, is prone to a change, the electrode’s preparation procedure was followed step by step by both X-ray diffraction and FT-IR measurements. X-ray patterns (Figure 4a) imply that the structure of the VOPO_4_·2H_2_O powder is stable on grinding in a mortar, drying at 120 °C, and ageing in the air for 7 months. However, mixing the VOPO_4_·2H_2_O with carbon black causes the appearance of new 2θ peaks at around 12.40°, 25°, and 29.44° in close proximity to the 001, 002, and 200 reflections, respectively (Figure 4b).

These peaks can be attributed to a new phase of the smaller *c* parameter, probably formed as a consequence of the partial loss of the intercalated water due to the hydrophilic nature of carbon additive (PBX51). On further addition of the dissolved PVDF in NMP to the mixture of the VOPO_4_·2H_2_O and carbon black, an abrupt disorder of the material’s structure can be noticed. Additional X-ray diffraction measurements of the mixture with NMP solvent alone showed that essentially NMP is responsible for the collapse of the structure due to its polarity and surface tension. The mechanism most probably follows the scheme for graphite exfoliation [34]; however, this time, the penetration of NMP between layers of VOPO_4_·2H_2_O causes the breakage of hydrogen bonding between crystal water and lattice oxygen, resulting in part of the water molecules leaving the structure. This gives rise to an X-ray pattern with very few peaks of much lower intensities, indicating both the decrease in crystallinity and the severe preferred orientation. Additionally, the swelling/exfoliation process cannot be ruled out [35]. A shift in the most intense peak to a higher angle implies a decrease in the interlayer distance (around 5.8 Å). In addition, FTIR measurements confirmed both the partial loss of water molecules and the presence of NMP molecules. Another binder was utilized, Nafion 5 wt% solution in a mixture of lower aliphatic alcohols and water. It turned out that the Nafion solution also alters the pristine structure (Figure 4b), but in a different way. This time, well-defined, intense new peaks are observed, while the peaks of the pristine structure, VOPO_4_·2H_2_O, completely vanished. This new pattern entirely matches the pattern of the H_x_VOPO_4_·2.33H_2_O phase, which has a partly reduced vanadium and whose structure can be described in the *P*2_1_/*c* monoclinic space group [35]. The lattice parameters of the new monoclinic phase are obtained by Le Bail fit [36,37] and amount to *a* = 7.4, *b* = 26.4, and *c* = 8.8 Å with an angle β = 106.6°. They are related to the lattice parameters of the pristine tetragonal phase by the equations *b*_m_ = $\sqrt{2}$·3*a*_t_ and *c*_m_ = $\sqrt{2}$*a*_t_ (where t and m refer to the tetragonal and monoclinic phases, respectively). These findings are in agreement with the results of Shpeizer et al. [35], who first reported the existence of this phase. Previously mentioned additional peaks that emerged in the XRD pattern of the mixture comprising VOPO_4_·2H_2_O and carbon black can be indexed as belonging to this reduced phase. To the best of our knowledge, none of the papers in the literature report similar phenomena.

**Figure 5:**
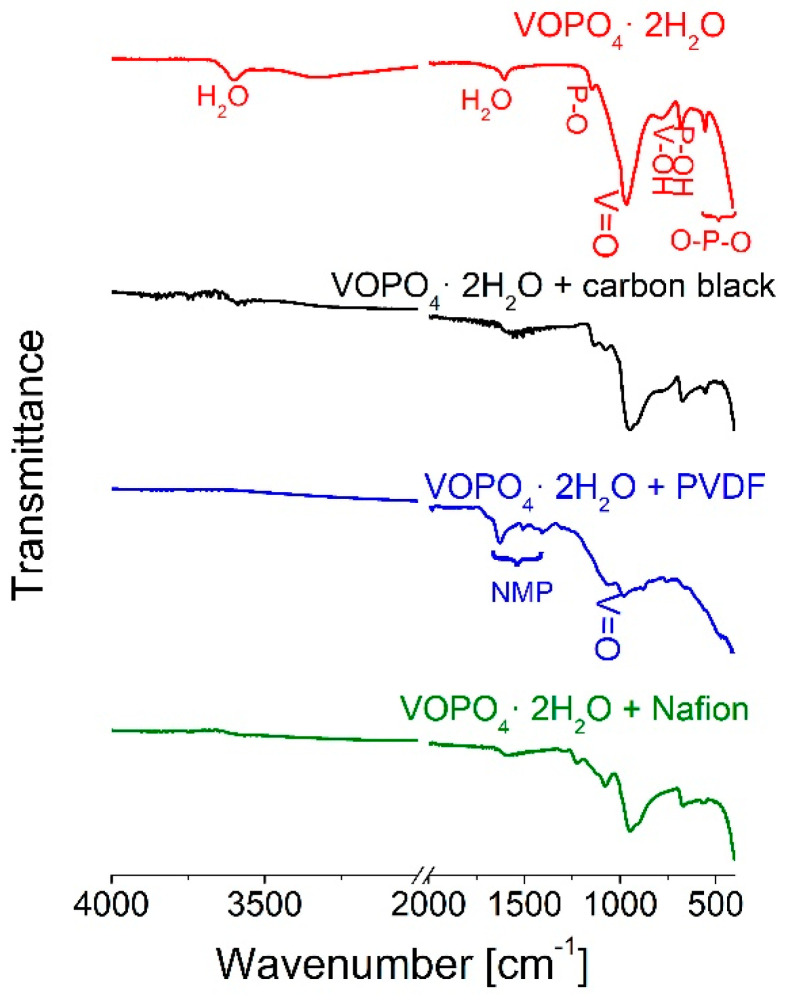
Comparison of FTIR spectra of the VOPO_4_·2H_2_O and its mixtures with carbon black, carbon black and PVDF, and carbon black and Nafion.

Figure 5 shows continuous changes in the band features of the infrared spectra. The bands attributed to water molecules (at 3600, 3300, and 1600 cm^−1^) decrease in intensity, even after mixing the pristine powder with carbon black. This is associated with lattice water expulsion, as assumed in the XRD analysis. The addition of a binder solution to this mixture causes a profound intensity decrease in the water bands (when Nafion is a binder) or their full disappearance (when PVDF is a binder). The spectrum of the PVDF-containing mixture underwent the greatest changes, with the bands of the parent phase subsiding while the new bands rise. These bands correspond to NMP [38], which can be interpreted as pyrrolidone being incorporated into the crystal structure, most likely as a partial replacement of the water molecules in the interlayer space. Bands that correspond to the vibration of the PO_4_ group are slightly red-shifted, implying bond elongation due to a replacement of water molecules with solvent molecules.

### 3.3. Electrochemical Properties

**Figure 6:**
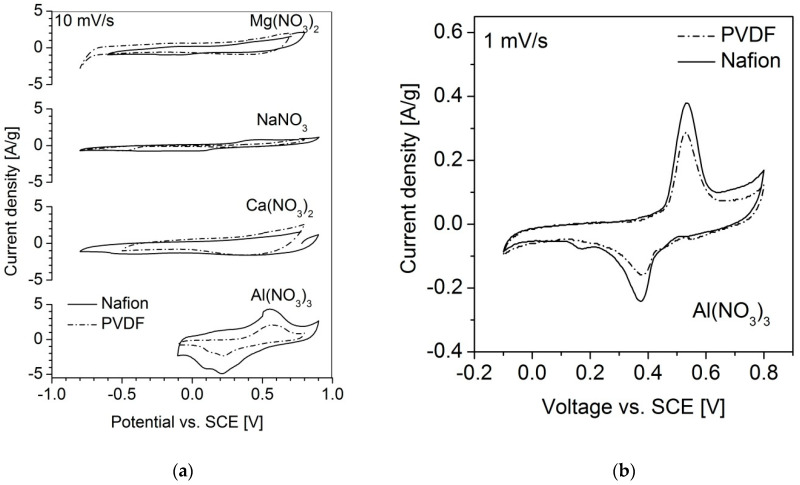
(**a**) Cyclic voltammograms of the composite electrode comprising VOPO_4_·2H_2_O, carbon black, and a binder (PVDF or Nafion) in NaNO_3_-, Ca(NO_3_)_2_-, Mg(NO_3_)_2_-, and Al(NO_3_)_3_-saturated aqueous solutions at scan rate of 10 mVs^−1^. (**b**) Cyclic voltammograms of the composite electrode comprising VOPO_4_·2H_2_O, carbon black, and a binder (PVDF or Nafion) in Al(NO_3_)_3_-saturated aqueous solution at scan rate of 1 mVs^−1^.

Cyclic voltammetry measurements were used to study the electrochemical response of VOPO_4_·2H_2_O in various aqueous electrolytes, as displayed in Figure 6. Figure 6a presents the cyclic voltammograms (CV) at a 10 mVs^−1^ scan rate of an electrode immersed in NaNO_3_, Ca(NO_3_)_2_, Mg(NO_3_)_2_, and Al(NO_3_)_3_ solutions. An interesting feature is that the CV curves profiles vary not only by the variation of electrolyte but also with the binder used in electrode preparation. It can be noticed that the best electrochemical response was obtained when the cycling was performed in an aluminium nitrate solution.

**Figure 7:**
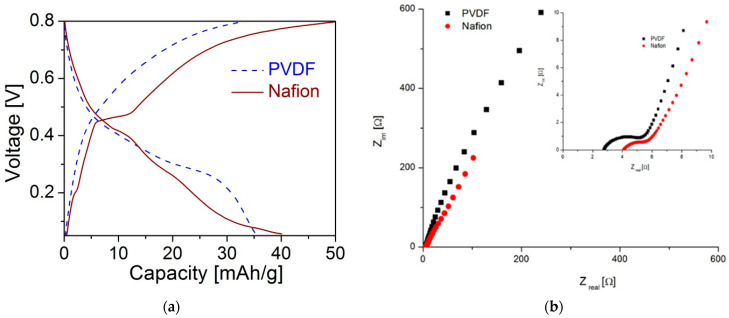
(**a**) Galvanostatic curves obtained for the composite electrodes comprising VOPO_4_·2H_2_O, carbon black, and a binder (PVDF or Nafion) in Al(NO_3_)_3_ saturated aqueous solution; applied current: 20 mA/g. (**b**) The corresponding Nyquist plots for two cells with these electrodes. The inset is enlarged high-frequency region.

The cyclic voltammograms obtained in the Al(NO_3_)_3_ solution are characterized by a larger enclosed area and fairly defined redox peaks of higher current intensities. These peaks are more pronounced at a lower scan rate (Figure 6b) and can be ascribed to V^+5^/V^+4^ redox reactions on the basis of their position (~0.5 V vs. SCE) [39]. Galvanostatic charge–discharge tests in Al(NO_3_)_3_ solution are shown in Figure 7a. The continuous sloping curve profile indicates a homogeneous distribution of aluminium ions within the structure of the active material applied with PVDF binder. On the other hand, the stepped curve of the active material applied with Nafion binder indicates the structural transition of the host, i.e., possible changes in the crystallographic environment of vanadium and/or aluminium ion positions with consequent alternation of insertion/extraction voltage. However, for both electrodes (with PVDF or Nafion), the obtained capacity is smaller than the theoretical one, suggesting the sluggish charge transfer kinetics of the electrode.

Electrochemical impedance spectroscopy plots of the cells with Al(NO_3_)_3_-saturated aqueous solution (Figure 7b) are composed of a depressed semicircle at high frequencies (inset of Figure 7b), followed by an inclined line. The smaller radius of the semi-circle noticed for an electrode made with Nafion implies smaller charge transfer resistance. The structural changes created during the electrode’s preparation with Nafion binder alleviate charge transfer.

In contrast to Al(NO_3_)_3_, the CV curves (Figure 6a) recorded in the other three electrolytes (NaNO_3_, Ca(NO_3_)_2_, and Mg(NO_3_)_2_) exhibit quasi-rectangular shapes with broad peaks and large polarization, implying pseudocapacitive behaviour without noticeable redox activity. This can be interpreted that in the case of NaNO_3_, Ca(NO_3_)_2_, or Mg(NO_3_)_2_ electrolytes there are no intercalation reactions of a certain cation (Na^+^, Ca^2+^, or Mg^2+^) within the structure. Various factors related to the structure and to the electrolytes can be a cause of such differences, for example, the difference in the ionic radii of the cations [40], the pH value of the electrolytes, ionic conductivity, and the viscosity of the electrolyte [41,42].

## 4. Conclusions

VOPO_4_·2H_2_O, synthesized by the sonochemical method, was investigated as a cathode material in aqueous cells. The crystal structure refinement confirmed crystallization in the P4/*nmm*Z space group. The structure of the synthesized powder is stable upon ageing in air, grinding in a mortar, and heating to 120 °C. During the preparation of the electrode, it was found that the binder influences the structure of the active material, which has not been recorded in the literature so far. Namely, the use of a conventional binder PVDF in NMP solution degrades the structure and lowers its crystallinity, while the use of Nafion solution causes the rearrangement of the atoms in a new crystal form that can be described as a monoclinic *P*2_1_/*c* space group. The cycling voltammetry measurements evidenced electrochemical activity with a pronounced V^5+^/V^4+^ redox pair in Al(NO_3_)_3_ solution, regardless of the binder used. In contrast, CV curves recorded in NaNO_3_, Ca(NO_3_)_2_, and Mg(NO_3_)_2_ solutions exhibited quasirectangular shapes, implying pseudocapacitive behaviour. The study reveals new insights in the relation between binder and active material, often considered as mutually non-interacting.

## Figures and Tables

**Table 1:** The main results of the refinement in the space group P4/*nmm*Z.

Lattice parameters (Å)	a = 6.2138 (9) b = 6.2138 (9) c = 7.4140 (10)
Primitive cell volume (Å^3^)	V = 286.26 (9)
Mean crystallite size (nm)	280 (20)
Microstrain (%)	0.38 (2)

**Table 2:** Fixed and refined fractional atomic coordinates.

Fractional Coordinates	x	y	z	B [Å^2^]
P1 (2b)	0.75	0.25	0.5	2.5
V (2*c*)	0.25	0.25	0.4092 (5)	0.5
O1 (8*i*)	0.25	0.941 (2)	0.3809 (8)	0.4
O2 (2*c*)	0.25	0.25	0.091 (2)	2.1
O3 (2*a*)	0.75	0.25	0	2.1
O4 (2*c*)	0.25	0.25	0.631 (1)	2.1

**Table 3:** Selected bond distances and polyhedral distortions.

M–O Bond	Bond Length [Å]
P–O1 × 4	1.4804 (97)
P–O3 × 2	3.7070 (5)
(P–O1) _aver._	1.4804 (97)
PO_4_ distortion	0
V–O1 × 4	1.930 (11)
V–O2	2.357 (13)
V–O4	1.644 (11)
(V–O) _aver._	1.954
VO_6_ distortion	1.1 × 10^−2^

## Data Availability

The raw data required to reproduce these findings cannot be shared at this time as the data also form part of an ongoing study.
